# Lifestyle Behavioral Self-Care Practices and Their Determinants Among Type 2 Diabetes Patients Attending a Non-communicable Disease Clinic in Agra District: A Cross-Sectional Study

**DOI:** 10.7759/cureus.98857

**Published:** 2025-12-09

**Authors:** Dhiraj Singh, Himalaya Singh, Manisha M Nagargoje, Purnoor Kaur, Sarvesh Kumar, Suvit S Sengar

**Affiliations:** 1 Community Medicine, Sarojini Naidu Medical College, Agra, IND

**Keywords:** diabetes mellitus management, diabetes self-care, lifestyle behavior, lifestyle behavior self-care practices, non-communicable disease, non-communicable disease clinic, summary of diabetic self-care activities (sdsca) tool, type 2 diabetes mellitus

## Abstract

Background: Type 2 diabetes (T2D) requires sustained self-care practices to achieve optimal glycemic control and prevent complications. In India, adherence to diabetes self-management remains suboptimal and is influenced by various sociodemographic and lifestyle behavioral factors. Therefore, the present study aimed to assess lifestyle-related self-care practices among patients with T2D attending a non-communicable disease (NCD) clinic in Agra District and to examine their association with sociodemographic characteristics.

Materials and methods: A cross-sectional study was conducted among 340 patients with T2D aged 30-60 years who attended the NCD clinic at District Hospital in Agra from May to October 2024. Using systematic random sampling, data were collected through a semi-structured questionnaire based on the modified Summary of Diabetes Self-Care Activities tool, adapted to the local context. Lifestyle self-care scores were calculated across four domains: diet, physical activity, foot care, and addiction. Logistic regression was used to identify factors associated with self-care scores.

Results: The mean age of participants was 51.3 ± 10.6 years, and 56.5% were female. Overall, 73.2% demonstrated moderate self-care, while 21.2% had poor self-care. Lack of daily foot inspection was reported by 77.9%, and only 2.1% engaged in adequate physical activity. Significant predictors of poor self-care included male gender, lower educational attainment, and unemployment (p < 0.05). Urban residence and higher occupational status were associated with better self-care scores.

Conclusions: Self-care practices were moderately adhered to, with particularly low levels of physical activity and medication adherence. Strengthening the communication of diabetes self-management education and support should be prioritized to promote sustained lifestyle modification and proactive self-care, especially among men, individuals with lower education, and those who are unemployed.

## Introduction

Self-care practices play a pivotal role in the management of type 2 diabetes (T2D). As a chronic condition, T2D requires ongoing attention to lifestyle, diet, and medication adherence to prevent complications and maintain optimal glycemic control. Effective self-care is essential for improving quality of life and reducing the risk of complications such as cardiovascular disease, neuropathy, and nephropathy [1]. Understanding the determinants of self-care behaviors among individuals with T2D can inform healthcare strategies to improve patient outcomes. Self-care in T2D encompasses a range of activities, including blood glucose monitoring, adherence to prescribed medications, physical activity, healthy eating, and regular foot care [2,3].

A variety of factors influence self-care behaviors among people with T2D. These determinants broadly fall into individual, interpersonal, and systemic domains. At the individual level, demographic factors such as age, gender, and education, along with psychological factors including self-efficacy and health literacy, play a substantial role [4]. Studies have shown that higher self-efficacy is associated with better adherence to self-care behaviors, underscoring the importance of patients’ confidence in managing their condition [5]. Likewise, health literacy, the ability to obtain, process, and understand essential health information, is a critical determinant of effective diabetes self-management [6]. Interpersonal factors, including social support from family members, peers, and healthcare providers, further influence self-care practices. Evidence suggests that individuals who receive emotional and practical support are more likely to engage in health-promoting behaviors [7]. The role of healthcare providers in delivering tailored education and guidance is equally important, as diabetes self-management education and support programs have been shown to enhance patients’ self-care capabilities significantly [8].

Given the multifaceted nature of these determinants, a comprehensive understanding of the factors influencing self-care is crucial for designing effective interventions. Tailored approaches that address individual, interpersonal, and systemic barriers are necessary to promote sustainable self-care practices among individuals with T2D [9]. In India, the Ministry of Health and Family Welfare has launched a national program to prevent and control non-communicable diseases (NCDs), including diabetes, through the "National Programme for Prevention and Control of Non-Communicable Diseases" [10]. While the program emphasizes early diagnosis, it places limited focus on self-care practices among diabetic patients. Given the high reliance on the public healthcare system, understanding existing self-care behaviors is essential yet remains underexplored in Indian settings [10-12].

Against this backdrop, the present study assessed lifestyle-related self-care practices, including diet, physical activity, and foot care, among adults with T2D attending an NCD clinic and examined their association with sociodemographic characteristics. The findings will generate evidence to support program managers in understanding the epidemiological distribution of lifestyle self-care practices in diabetes management and identifying patient-level barriers to their adoption.

## Materials and methods

Study design and setting

The present cross-sectional study was conducted under the Department of Community Medicine, Government Medical College, Agra, to assess self-care practices among individuals with T2D attending the NCD clinic (Table 1). The single NCD clinic in Agra, located at District Hospital, was selected as the study site.

**Table 1:** Parameters for evaluating lifestyle behavioral self-care practices in T2D patients attending an NCD clinic T2D: type 2 diabetes, NCD: non-communicable disease

Domain	Parameters	Minimum	Maximum
Physical activity	Level of physical activity	2	8
	Weekly physical activity (minutes)		
	Sedentary behavior		
Diet	Daily fruit consumption	1	4
	Daily meal frequency		
Foot care	Daily foot inspection	0	1
Addiction	Alcohol consumption in the last month	0	4
	History of alcohol consumption		
	Smoking tobacco usage		
	Chewing tobacco usage		
Total		3	17

Study population

The study population comprised all T2D patients who attended the NCD clinic at District Hospital in Agra between May and October 2024. Adults aged ≥30 years who had been diagnosed with T2D and were undergoing diabetes treatment for at least one year were included in the study [11]. Patients with other types of diabetes or gestational diabetes, those with cognitive impairment, and those unable or unwilling to participate were excluded.

Sampling size and sampling procedure

The sample size was calculated using the single-population proportion formula. Assuming a 5% margin of error, a 95% confidence interval (CI), a 5% non-response rate, and using the proportion of diabetes self-care practice (46.7%) reported in a previous study from the Tigray region [12], the final sample size was determined to be 340. Study participants were selected using systematic random sampling. Each OPD day at the NCD clinic, the first participant was selected by lottery, and every 3rd eligible participant thereafter was included, based on an estimated daily attendance of approximately 60 diabetes patients. This process continued until the required sample size was achieved.

Data collection procedure

Data were collected daily at the NCD clinic by a single interviewer using the sampling procedure until the required sample size was achieved. A pre-tested, semi-structured, interviewer-administered questionnaire was used to collect information from diabetic patients attending the clinic for routine medical check-ups.

The questionnaire consisted of two sections. The first section collected sociodemographic information, including age, gender, marital status, education, and socioeconomic status, classified using the modified BG Prasad scale, updated for February 2024 [13]. All data were recorded using standardized formats, and participants were assigned numerical identifiers to ensure anonymity and confidentiality.

The second section assessed lifestyle-related self-care practices specific to diabetes across four domains: physical activity, diet, foot care, and addiction. These domains were selected based on established evidence linking them to improved glycemic outcomes [14].

Ten pre-tested items corresponding to these domains were included in a principal component analysis, which yielded a four-component structure. Sampling adequacy was acceptable (KMO = 0.587), and Bartlett’s test of sphericity was significant (p < 0.001), confirming that the data were suitable for factor extraction. The extracted components demonstrated adequate communalities and clear, interpretable loadings.

Each item was scored on a scale from 0 to 4, where a score of "0" indicated no engagement and higher scores (up to 4) reflected greater adherence to the specific self-care behavior. This scoring approach enabled more nuanced quantification of adherence than a simple binary classification. Item scores were summed across the four domains to generate a cumulative self-care score for each participant.

Total scores were then dichotomized at the sample mean to classify participants into two categories: good self-care (scores above the mean) and poor self-care (scores at or below the mean). This method provided a standardized and interpretable classification for comparing self-care practices within the study population.

Data management and analysis

To ensure data quality and questionnaire alignment with the study objectives, a pretest was conducted on 5% of the total sample of patients attending diabetes clinics at a tertiary-level teaching hospital that was not selected as the study site. During data management, storage, and analysis, all collected data were thoroughly checked for accuracy and consistency. Data were entered into Microsoft Excel (Microsoft Corp., Redmond, WA, USA) and analyzed using Epi-Info version 7.2.6.0 (Centers for Disease Control and Prevention, Atlanta, GA, USA). Categorical variables were presented as proportions, while continuous variables were summarized using mean, standard deviation (SD), median, and interquartile range (IQR). Univariate and multivariate logistic regression analyses were performed to identify associated risk factors, and adjusted odds ratios (AORs) with 95% CI were reported. A p-value of <0.05 was considered statistically significant.

Study variables

Sociodemographic factors included age, sex, place of residence, educational status, occupation, marital status, religion, and estimated monthly income. Behavioral factors included a history of smoking, alcohol consumption (lifetime use), and engagement in physical activity.

Ethical considerations

Ethical approval for the study was obtained from the Institutional Review Board of S. N. Medical College, Agra (approval number: SNMC/IEC/2024/204, dated February 2, 2024). Permission to conduct the research was also secured from the relevant health institution. Written informed consent was obtained from all participants, and anonymity was ensured by assigning non-identifiable codes.

## Results

Sociodemographic characteristics of study participants

Table 2 shows that among the 340 study participants, the mean age was 51.3 ± 10.6 years. The largest proportion (64.6%) was aged 46-60 years, followed by 22.1% aged 30-45 years and 13.3% aged 60 years and older. Females comprised 56.5% of the sample, and males 43.5%, with 88.7% of participants married. Regarding educational status, 39.5% had completed high school, while 34.7% were illiterate. Most participants (75.3%) resided in urban areas. In terms of family leadership, 57.6% of households were headed by the participant themselves ("self"), and the spouse headed 39.1%. According to the modified BG Prasad socioeconomic classification (2024), 30.5% of participants belonged to the middle class, 28.5% to the lower middle class, and 21.2% to the upper middle class, while the upper class and lower class each accounted for less than 12% of the population.

**Table 2:** Sociodemographic characteristics of T2D patients attending the NCD clinic at District Hospital T2D: type 2 diabetes, NCD: non-communicable disease

Sociodemographic characteristics	Category	Frequency n = 340 (%)
Mean age in years	51.3 ± 10.6	
Sex	Female	192 (56.5)
	Male	148 (43.5)
Age groups in years	30-45	75 (22.1)
	46-60	219 (64.6)
	>60	46 (13.3)
Married status	Single/divorced/widowed	38 (11.3)
	Married	302 (88.7)
Educational status	Illiterate	118 (34.7)
	Up to high school	134 (39.5)
	Intermediate and above	88 (25.8)
Residence	Rural	84 (24.7)
	Urban	256 (75.3)
Head of family	Self	196 (57.6)
	Spouse	133 (39.1)
	Others	11 (3.3)
Occupation	Employed	51 (15)
	Unemployed	289 (85)
Socioeconomic status (modified BG Prasad socioeconomic classification)	Upper	28 (8.3)
	Upper middle	72 (21.2)
	Middle	104 (30.5)
	Lower middle	97 (28.5)
	Lower	39 (11.5)

Lifestyle behavior, self-care practices, and their association with sociodemographic characteristics among study participants

**Figure 1:**
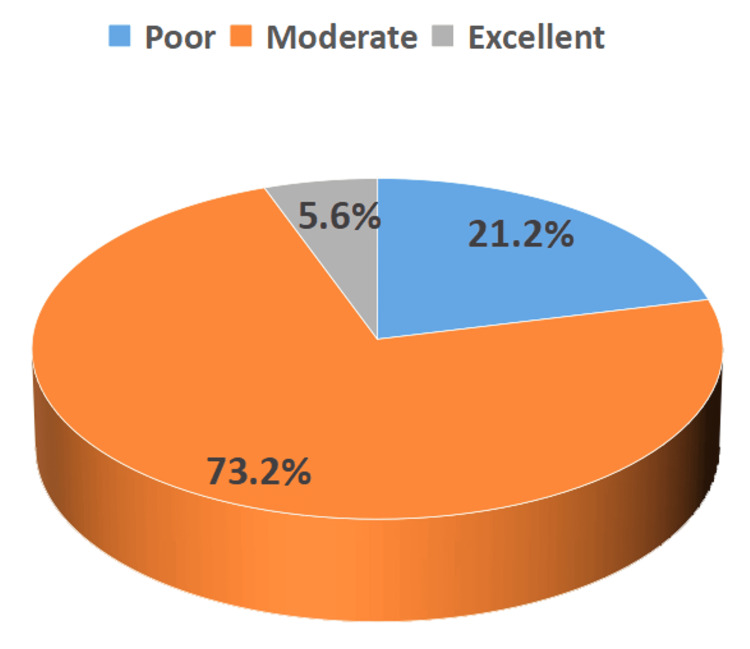
Distribution of lifestyle behavior self-care score among T2D patients attending the NCD clinic at District Hospital As per the Summary of Diabetes Self-Care Activities questionnaire. Poor self-care practices: <50%, moderate self-care practices: 50-70%, excellent self-care practices: >70% T2D: type 2 diabetes, NCD: non-communicable disease

Among the 340 study participants, the majority (73.2%) demonstrated moderate self-care practices (scores between 50 and 70%). In contrast, only 5.6% exhibited excellent self-care, and 21.2% fell into the poor self-care category (scores <50%), as illustrated in Figure 1. Table 3 shows that the overall mean self-care score was 7.45 ± 1.49, with a range of 3-17, indicating suboptimal engagement in self-care among the surveyed T2D patients.

**Table 3:** Lifestyle behavioral self-care practices among T2D patients attending the NCD clinic at District Hospital T2D: type 2 diabetes, NCD: non-communicable disease

Lifestyle behavior self-care parameters (study participants = 340)	Score 0 response (n, %)	Score 1 response (n, %)	Score 2 response (n, %)	Score 3 response (n, %)	Score 4 response (n, %)
Level of physical activity (score 0 - mild activity/walking, score 2 - vigorous activity)	338 (99.4)	-	2 (0.6)	-	-
Weekly physical activity (score 1 - <75 minutes, score 2 - 75-150 minutes, score 3 - >150 minutes)	-	331 (97.4)	7 (2.1)	2 (0.6)	00 (0)
Sedentary behavior (score 0 - yes, score 1 - no)	265 (77.9)	75 (22.1)	-	-	-
Daily fruit consumption (score 0 - none, score 1 - ≥1 serving)	271 (79.7)	69 (20.3)	-	-	-
Daily meal frequency (score 1 - 3 meals/day, score 2 - 3-5 meals/day, score 3 - >5 meals/day)	-	332 (97.6)	1 (0.3)	7 (2.1)	-
Daily foot Inspection (score 0 - no, score 1 - yes)	76 (22.4)	264 (77.6)	-	-	-
Alcohol consumption in the last month (score 0 - yes, score 1 - no)	39 (11.5)	301 (88.5)	-	-	-
History of alcohol consumption (score 0 - yes, score 1 - no)	52 (15.3)	288 (84.7)	-	-	-
Smoking tobacco usage (score 0 - yes, score 1 - no)	47 (13.8)	293 (86.2)	-	-	-
Chewing tobacco usage (score 0 - yes, score 1 - no)	98 (28.8)	242 (71.2)	-	-	-
Mean self-care score = 7.45 + 1.49, min = 3, max = 17, range = 14					

Regarding physical activity, almost all participants (99.4%) reported only mild activity or walking, with a negligible proportion (0.6%) engaging in vigorous activity. Most participants (97.4%) performed less than 75 minutes of physical activity per week, substantially below the recommended 150 minutes of moderate-intensity aerobic activity; only 0.6% met the 150-300 minutes target, and none exceeded 300 minutes. Sedentary behavior was highly prevalent, reported by 77.9% of participants.

Dietary data showed that 79.7% of participants consumed no servings of fruit daily, indicating inadequate intake of essential micronutrients and fiber. While 97.6% adhered to three meals per day, a small proportion (2.1%) consumed more than five meals per day. Foot care practices were suboptimal, with 22.4% of participants not performing daily foot inspections, placing them at risk for diabetic foot complications.

Regarding substance use, 88.5% reported no alcohol consumption in the past month, and 84.7% had no history of alcohol use; however, 11.5% were current drinkers, and 15.3% had a history of alcohol consumption. Tobacco use was notable, with 13.8% smoking and 28.8% using smokeless tobacco.

As shown in Table 4, gender (female), higher educational status (intermediate and above), and employment were significantly associated with better self-care practices (p = 0.000 for gender and education, p = 0.019 for employment).

**Table 4:** Association between sociodemographic characteristics and lifestyle behavioral self-care practices among T2D patients attending the NCD clinic at District Hospital COR: crude odds ratio, CI: confidence interval, T2D: type 2 diabetes, NCD: non-communicable disease

Sociodemographic characteristics N = 340		Self-care practices			COR (95% CI)	p-value
		Poor self-care score <7.0		Good self-care score ≥7.0		
Age	30-45 years	16 (21.3)		59 (78.7)	1	0.052
	46-60 years	53 (24.2)		166 (75.8)	1.177 (0.625-2.217)	
	>60 years	3 (6.7)		42 (93.3)	0.263 (0.072-0.962)	
Sex	Male	53 (35.8)		95 (64.2)	1	<0.001
	Female	19 (9.9)		173 (90.1)	0.197 (0.110-0.352)	
Educational status	Up to primary school	4 (10.5)		34 (89.5)	1	0.02
	Up to high school	15 (13.0)		100 (87.0)	1.275 (0.396-4.106)	
	Intermediate and above	53 (28.3)		134 (71.7)	3.362 (1.137-9.937)	
Residential status	Rural	24 (28.9)		59 (71.1)	1	0.031
	Urban	47 (18.4)		209 (81.6)	0.533 (0.313-0.978)	
Head of the family	Self	55 (28.1)		141 (71.9)	1	<0.001
	Spouse/other	17 (11.8)		127 (88.2)	0.343 (0.189-0.622)	
Occupation	Employed	2 (3.9)		49 (96.1)	1	0.019
	Unemployed	70 (24.2)	219 (75.8)		7.831 (1.857-33.029)	

The multivariate logistic regression analysis presented in Table 5 identified several key sociodemographic factors independently associated with poor self-care among patients with T2D. Educational status emerged as a significant predictor (Wald = 12.239, p = 0.002), with participants having "intermediate and above" education showing 3.36 times higher odds of poor self-care compared to those with "up to primary school" education (aOR = 3.362; 95% CI: 1.137-9.937; p = 0.028). Unemployment was also a significant risk factor, with unemployed individuals having approximately six times the odds of poor self-care compared with employed participants (aOR = 6.066; p = 0.019). Female gender was protective, with females having significantly lower odds of poor self-care than males (aOR = 0.209; p < 0.001).

**Table 5:** Association of sociodemographic predictors with poor lifestyle behavioral self-care practices among T2D patients attending the NCD clinic at District Hospital CI: confidence interval, aOR: adjusted odds ratio, T2D: type 2 diabetes, NCD: non-communicable disease

Variables									95% CI	
		B	S.E.	Wald	Df	Sig.	aOR	Lower		Upper
Age										
	30-45 years	-	-	4.444	2	0.108	-	-		-
	46-60 years	0.168	0.376	0.198	1	0.656	1.182	0.566		2.472
	>60 years	-1.21	0.710	2.887	1	0.089	0.299	0.074		1.204
Sex (male vs. female)		-1.57	0.417	14.16	1	0.000	0.209	0.092		0.472
Residential status (rural vs. urban)		-0.513	0.337	2.313	1	0.128	0.599	0.309		1.160
Educational status										
	Up to primary school	-	-	17.503	2	0.000	-	-		-
	Up to high school	1.971	0.587	11.286	1	0.001	7.180	2.273		22.678
	Intermediate and above	1.100	0.354	9.661	1	0.002	3.004	1.501		6.012
Head of the family (self vs. others)		-0.151	0.430	0.124	1	0.725	0.860	0.370		1.996
Occupation (employed vs. unemployed)		1.803	0.769	5.495	1	0.019	6.066	1.344		27.384
Constant		-3.300	0.988	11.150	1	<0.001	0.37	-		-

Conversely, age (p = 0.108), residential status (p = 0.128), and head-of-household status (p = 0.725) were not independently associated with poor self-care in this model. Notably, older age groups (46-60 years and >60 years) were less likely to engage in moderate or excellent self-care than the youngest age group (<30 years), highlighting an important demographic for targeted interventions. Overall, males had significantly lower odds of exhibiting moderate or excellent self-care compared to females.

## Discussion

The present study aimed to assess lifestyle and self-care practices among patients with T2D attending the NCD clinic. We found that while 73.2% of participants demonstrated moderate self-care scores, 21.2% fell into the poor self-care category. However, total scores may obscure specific gaps in patient care. Notably, only 2.1% of participants met the recommended physical activity levels of 75-150 minutes per week, which is concerning given that both Colberg et al. [12] and the WHO [13] emphasize regular exercise as essential for glycemic control and cardiovascular health in T2D.

Foot care was another area of significant concern. In our study, 77.6% of participants did not perform daily foot inspections. As noted by Boulton et al. [15], neglecting daily foot checks represents a missed opportunity to prevent complications, particularly in the context of diabetic neuropathy. This finding aligns with a study from Eastern India by Mitra et al. [16], which reported that 79% of patients had poor knowledge regarding foot care. Dietary practices were similarly suboptimal, with only 20.3% of participants consuming the recommended 400 g of fruits and vegetables per day, as advised by the WHO [17]. This finding is consistent with the Chennai Urban Rural Epidemiology Study by Radhika et al. [18], which identified cost and misconceptions as common barriers to adequate fruit and vegetable intake among Indian patients.

To explore factors influencing self-care, we dichotomized the "self-care score" (range: 3-13) using a mean split (cutoff ≥7.0) to classify participants as having either "good" or "poor" self-care. This approach was chosen because the data are non-normally distributed and allows the calculation of odds ratios for associated risk factors. This methodology aligns with recent diabetes studies, such as Dedefo et al. [19], and is consistent with the scale guidelines from Toobert et al. [14], facilitating clinically meaningful interpretation of the results.

Our multivariate analysis identified male gender, lower education, and unemployment as significant predictors of poor self-care. The association between lower education and suboptimal self-care is likely mediated by reduced health literacy, which can make it more difficult for patients to comprehend and adhere to complex instructions regarding diet and medication. These findings align with the Lancet Commission on Diabetes framework proposed by Chan et al. [20], which identifies economic instability as a key driver of inadequate diabetes management. Similarly, the ICMR-INDIAB study by Anjana et al. [21] demonstrated that diabetes outcomes in India are closely linked to socioeconomic status. Interestingly, we did not observe significant differences in self-care across religious groups, suggesting that socioeconomic factors, such as education and employment, have a stronger influence on health behaviors than cultural background, a trend also reported by Pradeepa et al. [22].

The study has several strengths, including its robust cross-sectional design and use of systematic random sampling, which helped minimize selection bias. However, reliance on self-reported data introduces potential recall and social desirability bias. The dichotomization of the self-care score simplified data collection and accommodated participants with varying levels of health literacy, albeit at the cost of some statistical precision. This binary format, however, reduced respondent burden and likely improved reporting accuracy. The tool, adapted from the Summary of Diabetes Self-Care Activities, was pretested to ensure clarity and contextual relevance. Limitations include the single-center setting, which may overrepresent urban patients and limit generalizability, and the exclusive focus on sociodemographic variables without accounting for clinical or psychosocial determinants.

## Conclusions

Attendance at the NCD clinic has not translated into adequate adherence to lifestyle-related self-care. Current education on key domains such as physical activity and foot care appears insufficient, particularly for high-risk groups, including men and unemployed individuals. Clinical strategies should move beyond passive knowledge dissemination toward targeted behavioral interventions that address these non-pharmacological aspects of diabetes management. Integrating regular diabetes self-care education with mandatory knowledge assessment during follow-up visits into primary care services may enhance patient comprehension and improve adherence.

## References

[1] Khosravizadeh O, Ahadinezhad B, Maleki A, Yousefy S, Momeni Z (2024). Diabetes self-care activities among patients with type 2 diabetes: a systematic review and meta-analysis. Int J Diabetes Dev Ctries.

[2] Martínez N, Connelly CD, Pérez A, Calero P (2021). Self-care: a concept analysis. Int J Nurs Sci.

[3] (2008). AADE7 self-care behaviors. Diabetes Educ.

[4] Joshi J, Patel P, Gandhi S, Patel N, Chaudhari A (2022). Factors influencing adherence to self-care practices among patients of type 2 diabetes mellitus from Saurashtra region of Gujarat: a conclusive research. J Family Med Prim Care.

[5] D MS, AS NM (2018). Self-efficacy impact adherence in diabetes mellitus. Diabetes Updates.

[6] Nutbeam D (2008). The evolving concept of health literacy. Soc Sci Med.

[7] Kalra S, Jena BN, Yeravdekar R (2018). Emotional and psychological needs of people with diabetes. Indian J Endocrinol Metab.

[8] Powers MA, Bardsley J, Cypress M (2015). Diabetes self-management education and support in type 2 diabetes: a joint position statement of the American Diabetes Association, the American Association of Diabetes Educators, and the Academy of Nutrition and Dietetics. J Acad Nutr Diet.

[9] Hood KK, Hilliard M, Piatt G, Ievers-Landis CE (2015). Effective strategies for encouraging behavior change in people with diabetes. Diabetes Manag (Lond).

[10] Ministry of Health and Family Welfare (2023). Ministry of Health and Family Welfare. National Programme for Prevention and Control of Non-Communicable Diseases: Operational Guidelines. New Delhi: Government of India. National Programme for Prevention and Control of Non-Communicable Diseases: Operational Guidelines.

[11] Nittoori S, Wilson V (2020). Risk of type 2 diabetes mellitus among urban slum population using Indian diabetes risk score. Indian J Med Res.

[12] Colberg SR, Sigal RJ, Yardley JE (2016). Physical activity/exercise and diabetes: a position statement of the American Diabetes Association. Diabetes Care.

[13] World Health Organization (2020). Who Guidelines on Physical Activity and Sedentary Behaviour. https://www.who.int/publications/i/item/9789240015128.

[14] Toobert DJ, Hampson SE, Glasgow RE (2000). The summary of diabetes self-care activities measure: results from 7 studies and a revised scale. Diabetes Care.

[15] Boulton AJM, Vileikyte L, Ragnarson-Tennvall G, Apelqvist J (2005). The global burden of diabetic foot disease. Lancet.

[16] Mitra S, Majumdar KK, Bhanja S (2023). Prevalence of diabetic foot ulcers and assessment of foot care knowledge and practice among patients attending diabetic clinic of a tertiary hospital of eastern India. Int J Community Med Public Health.

[17] (2018). Healthy diet. https://www.who.int/news-room/fact-sheets/detail/healthy-diet.

[18] Radhika G, Sudha V, Mohan Sathya R, Ganesan A, Mohan V (2008). Association of fruit and vegetable intake with cardiovascular risk factors in urban south Indians. Br J Nutr.

[19] Dedefo MG, Ejeta BM, Wakjira GB, Mekonen GF, Labata BG (2019). Self-care practices regarding diabetes among diabetic patients in West Ethiopia. BMC Res Notes.

[20] Chan JC, Lim LL, Wareham NJ (2020). The Lancet Commission on diabetes: using data to transform diabetes care and patient lives. Lancet.

[21] Anjana RM, Unnikrishnan R, Deepa M (2023). Metabolic non-communicable disease health report of India: the ICMR-INDIAB national cross-sectional study (ICMR-INDIAB-17). Lancet Diabetes Endocrinol.

[22] Pradeepa R, Anjana RM, Joshi SR (2015). Prevalence of generalized &amp; abdominal obesity in urban &amp; rural India--the ICMR-INDIAB Study (Phase-I) [ICMR- NDIAB-3]. Indian J Med Res.

